# Pharmacophore Directed Screening of Agonistic Natural Molecules Showing Affinity to 5HT_2C_ Receptor

**DOI:** 10.3390/biom9100556

**Published:** 2019-10-01

**Authors:** Ganesh Kumar Veeramachaneni, V B S C Thunuguntla, Maharshi Bhaswant, Michael L. Mathai, Jayakumar Singh Bondili

**Affiliations:** 1 Department of Biotechnology, Koneru Lakshmaiah Education Foundation (KLEF), Green Fields, Vaddeswaram, Guntur (Dt.) A.P. 522 502, India; ganesh.vgk55@gmail.com; 2 Institute of Health and Sport, Victoria University, Melbourne 3011, Australia; venkata.thunuguntla@live.vu.edu.au (V.B.S.C.T.); Michael.Mathai@vu.edu.au (M.L.M.); 3 Molecular Nanomedicine Research Unit, Centre for Nanoscience and Nanotechnology, Sathyabama Institute of Science and Technology, Chennai 600 119, India; cmaharshi@gmail.com

**Keywords:** 5HT_2C_ receptor, ZINC natural molecule database, pharmacophore modeling, glide, dynamic simulations

## Abstract

Obesity prevalence continues to be a foremost health concern across the globe leading to the development of major health risk conditions like type II diabetes, hyperlipidemia, hypertension and even cancers. Because of the deprived drug-based management system, there is an urgent need for the development of new drugs aiming at satiety and appetite control targets. Among the reported satiety signaling targets, 5HT_2C_ receptor plays a crucial role in decreasing appetite and has become a promising target for the development of anti-obesity drugs. Lorcaserin, a 5HT_2C_ receptor agonist and the only drug available in the market, was designed based on the receptor mechanism of action. Due to limited drug options available and considering the adverse drug effects of Lorcaserin, the development of new drugs which are highly specific toward the 5HT_2C_ target and with lesser side effects is essential. The present study is majorly focused on developing new 5HT_2C_ agonists through computational approaches like screening, docking, and simulation using Phase, QikProp, Glide and Desmond applications of the Schrodinger suite. Screening protocols resulted in eight best hit molecules with affinity for the receptor and among them, five hits displayed binding affinity toward the conserved residue Asp 134 of the receptor. The stability of the five molecules in complex with the 5HT_2C_ receptor was studied through molecular dynamic simulations. Three molecules, ZINC32123870, ZINC40312983 and ZINC32124535, maintained stable interactions with the Asp 134 residue throughout the 50 ns simulation run time. Further, due to the high sequence similarity seen among the receptors of 5HT2 family, the three potential hits were cross validated against other subtypes 5HT_2A_ and 5HT_2B_ of the 5HT2 family to determine the specificity of the molecules against the target. Among the three hits, ZINC32124535 was identified as the best potential hit based on the hydrogen bond interaction percentage with Asp residue [5HT_2A_ (Asp 155:60%); 5HT_2B_ (Asp155: No interaction); 5HT_2C_ (Asp 134:86%)]. The ZINC32124535 molecule produced one salt bridge and hydrogen bond interactions with Asp 134, alike the known drug Lorcaserin. Based on the results, ZINC32124535 was identified as the best potential hit against the 5HT_2C_ receptor.

## 1. Introduction

Obesity, an important threat to global public health in terms of prevalence and economic burden, demands a wide range of interventions from prevention to advance treatments. Obesity is primarily a condition involving excessive fat accumulation in the body and is associated with various health problems like developing type II diabetes, dyslipidaemia, fatty liver disease, osteoarthritis, coronary heart disease, hypertension, coronary artery disease and even cancers [1,2,3]. So far, several targets related to the central nervous system (CNS) like 5HT_2C_, NPY (NeuropeptideY), and AgRP (Agouti-related protein) etc., and the non-central nervous system like pancreatic lipase, leptin, ghrelin etc., [4,5] were reported as obesity targets. However, only pancreatic lipase (inhibitor) and 5HT_2C_ receptor agonists have gained importance in drug discovery compared to the other targets. To date, there are six Food and Drug Administration (FDA)-approved drugs available in the market. Among them, Orlistat (pancreatic lipase inhibitor) and Lorcaserin (5HT_2C_ receptor agonist) widely persisted with a weight loss of 3.0% and 3.0–3.6% per year, respectively, compared to placebo [6,7].

The present study is mainly focused on the 5HT_2C_ receptor, one of the obesity neuroreceptor targets mainly involved in satiety. The 5HT_2C_ receptor belongs to the 5HT_2_ family (serotonin), which is highly expressed in the central nervous system and targeted in multiple disorders such as obesity, eating disorders, depression, anxiety, obsessive-compulsive disorder, chronic pains, epilepsy, and erectile dysfunction [8,9,10,11,12,13,14,15,16,17,18]. The 5HT_2_ receptor family was further classified into three subtypes, namely 5HT_2A_, 5HT_2B_ and 5HT_2C_ with high sequence similarity, playing crucial roles in signal transduction pathways [19]. Due to the high sequence similarity within the subtypes, it has been a challenge to design drugs specifically targeting the 5HT_2C_ receptor.

Neural signaling related to food intake is controlled by ventromedial nucleus of hypothalamus (satiety center) and lateral hypothalamus (hunger center). The intake of food is simulated by agouti-related protein and neuropeptide Y, known as the stimulatory group, whereas cocaine and amphetamine-regulated transcript (CART) and pro-opiomelanocortin (POMC) neurons are considered as supressors of food intake [20]. The activation of 5HT_2C_ neurons of anorexigenic POMC activates the release of alpha-melanocyte-stimulating hormone (α-MSH). α-MSH further acts on the Melanocortin 4 receptor (MC4R) present in the para ventricular nucleus of hypothalamus, which promotes decrease in appetite [21]. Hence, the 5HT_2C_ receptor is considered to be a potent obesity target [22].

Lorcaserin ([1R]-8-chloro-2,3,4,5-tetrahydro-1-methyl-1H-3-benzazepine) is a known FDA-approved 5HT_2C_ receptor agonist [23] aimed to treat obesity by reducing hunger craving and suppressing appetite [24]. Many side effects like cardiovascular problems, nausea, vomiting, constipation, diarrhea, fatigue, upper respiratory tract infection, urinary tract infections, back pain, headache, dizziness and rash were reported by Lorcaserin usage [25,26]. Overall, the usage of this drug had minimal effects on body weight but had many side effects. Hence, there is a need to develop new generation drugs which can act specifically with lowered side effects. The current study analyzed pharmacophore-based screening of natural molecules targeted toward the 5HT_2C_ receptor and validated them against other subtypes for specificity via docking and molecular dynamic simulation studies.

## 2. Materials and Methods

### 2.1. Pharmacophore Hypotheses Generation and Screening

#### 2.1.1. Dataset Preparation

A total of 69 agonists from previous studies along with their known activity were considered in the development of the pharmacophore model [27]. All the molecules were sketched using the Chembio draw tool imported to Maestro. LigPrep module was used to prepare the molecules by applying OPLS_2005 force field at pH 7, followed by conformer generation using ConfGen of Macro-Model module with thorough sampling.

#### 2.1.2. Pharmacophore Hypotheses Generation and Validation

The pharmacophore modeling was performed using the PHASE module of Schrodinger suite [28]. The PHASE module develops the pharmacophore hypotheses from multiple ligands. The skeleton of pharmacophore involved six features: Hydrogen bond donor (D), hydrogen bond acceptor (A), hydrophobic region (H), negatively charged region (N), positively charged region (P), and aromatic rings (R). With the scoring parameters, the hypotheses were ranked based on the alignment against the active dataset. In addition, the quality of the hypothesis was assessed using parameters such as survival score, sites, vector, and selectivity scores. In this protocol, four out of five pharmacophore features were chosen as must match and other features with default settings. The hypothesis validation application from the PHASE module of Schrodinger suite was used to validate the hypotheses. To run the application, the active dataset and a decoy set were needed. The decoy set, comprising of 1000 molecules with a molecular weight of 360, was employed. The pharmacophore hypothesis validation was confirmed based on the number of actives retrieved from active dataset, percentage of actives resulted from decoy set and enrichment factor (EF).

#### 2.1.3. Screening of the ZINC Database

The ZINC natural compound database, containing nearly 140,311 compounds, was used in screening the agonists for the 5HT_2C_ receptor. These molecules were initially minimized using the Canvas 3D minimization tool from Schrodinger suite. The minimized natural molecule database was screened using advanced pharmacophore screening of the PHASE module [28] with validated pharmacophore hypotheses and hits acquired were ranked based on their fitness scores.

### 2.2. ADME Screening and PAINS

Drug-likeness of the hits obtained from the above screening filter was predicted by QikProp assessing pharmacological properties such as CNS, QPlogBB and Lipinski’s rule-of-five descriptors [29]. The central nervous system (CNS) property predicts the efficiency of the molecule in the central nervous system and ranges between +2 (active) and −2 (inactive). The predicted brain/blood (QPlogBB) range of −3.0 to 1.2 predicts the ability of the molecule to cross the blood brain barrier. The Lipinski’s rule-of-five property was used: Hydrogen bond donor ≤ 5, hydrogen bond acceptor ≤ 10, QPlogPo/w ≤ 5, molecular weight < 500 KDa. Compounds satisfying these properties were considered as drug–like compounds. The filtered molecules were further studied using ligand docking studies. A pan-assay interference compounds (PAINS) filter was also implied over screened molecules to remove false positives [30].

### 2.3. Receptors Preparation

Prior to the docking studies, 5HT_2_ family member’s 5HT_2A_, 5HT_2B_, and 5HT_2C_ receptors were prepared. For 5HT_2B_ and 5HT_2C_, crystal structures were available, whereas the 5HT_2A_ crystal structure was not available. Hence, to elucidate the 5HT_2A_ three-dimensional structure, Prime homology modeling application was opted. The 5HT_2C_ receptor crystal structure (PDB ID: 6BQG) was chosen as a template to build the 5HT_2A_ homology model. The first step involved in the homology modeling was sequence retrieval from the Uniprot database (ID: 28223) in FASTA format and importing into the modeling tool. Further, Protein Specific Iterated (PSI) BLAST algorithm (integrated in the application) [31] was performed against Protein Data Bank (PDB) [32] to identify the homologous protein structures and the template was selected based on the sequence similarity. Target and the template sequences were aligned using the ClustalW embedded in the module. Further, through SSpro program bundled in the application, the secondary structure of the receptor was predicted. Crystal protein conjugated ligands were not included during the structure modeling. In a similar way, the 5HT_2C_ homology model was also built, choosing the protein sequence (UniProtKB - P28335) and template PDB ID: 4NC3 for comparative study with the crystal structure. The crystal and modeled receptors were refined before proceeding to docking studies. The protein preparation wizard tool was used to refine the receptor and parameters used in refining the receptors were, adding hydrogens; creating disulphide bonds, zero order bonds, and metal atoms; converting any selenomethionines to methionines and desolvation by removing crystallized free water molecules beyond the 5 Å distance from the ligand [33]. Further, the hydrogen bonds in the receptor were optimized to repair overlapping hydrogens. Finally, force field OPLS_2005 was applied to minimize the receptor [34].

### 2.4. Ligand Docking Studies

Ligand docking was performed using the GLIDE module, following grid-based docking protocol. Once the binding site was analyzed, the receptor continued to generate a grid around the predicted active site to lock active amino acids inside the grid (Grid box size (x, y, z coordinates): Grid-center: 38.63, 31.17, 56.86; Inner-box: 10, 10, 10; and Outer-box: 27.39, 27.39, 27.39). Finally, hits were obtained after absorption, distribution, metabolism and excretion (ADME) filters and docked against the predicted active site of the modeled receptor using the Glide XP docking protocol [35]. The major settings involved in the docking studies were adding the receptors grid file in the receptor grid box, prepared ligands in the ligands tab, keeping precision to XP (extra precision) in the setting tab and returning all tab parameters to default. Based on the G-scores, molecules were tabulated.

### 2.5. Binding Free Energy Calculations Using Prime/MM-GBSA

Prime/MM-GBSA was employed to calculate the binding free energies of docked complexes. The pose viewer file of docked complexes with OPLS-AA force field in VSGB solvent model was used to calculate energies. Using the following equation, free energy of the complexes was calculated.

MMGBSA dG Bind = E_Complex_ − E_Receptor_ − E_Ligand_ (1)

where MMGBSA represents molecular mechanics energies combined with the generalized Born and surface area continuum solvation, dGbind represents the calculated relative free energy of both the ligand and receptor strain energy, E_complex_ represents the MM/GBSA energy of the minimized complex, E_receptor_ represents the mean MM/GBSA energy of protein (unbound, minimized) without ligand, and E_ligand_ represents the MM/GBSA energy of the ligand after removing it from the complex [36].

### 2.6. Molecular Dynamics Simulations

The 5HT_2C_ receptors in complex with the lead molecules were further refined by molecular dynamic simulations (MDS) using Desmond software [37,38] and their interaction consistency was studied. Initially, using the system builder application of desmond module, the modeled receptor was inserted into the pre-equilibrated palmitoyl-2-oleoyl-sn-glycero-3-phosphocholine (POPC) bilayer with default parameters and solvated with SPC (simple point-charge) water model in an orthorhombic periodic boundary box (Box size; distances (Å): a:10 × b:10 × c:10 and Angles: α:90^0^ × β:90^0^ × γ:90^0^). To neutralize the system, Cl^−^ ions were added depending on total charge of model along with 0.15 M salt concentration. The next step was minimizing the model obtained from the system builder by setting the maximum iterations to 2000. The remaining parameters were set to default in the minimization application. Finally, dynamic simulations were carried with the minimized model in NPT (isothermal–isobaric ensemble, Number of particles (N), Pressure (P) and Temperature (T)) ensemble, 300 K temperature and 1 bar pressure, relaxed using molecular dynamics application of the Desmond module. The molecular dynamic simulations (general sampling method) job was carried out for a period of 50 ns. From the obtained trajectory file, root-mean-square deviation (RMSD), root-mean-square fluctuation (RMSF) and H-bond interactions of the complex were analyzed. During simulations, especially in the RMSF graph generation, the amino acid numbers were shifted, i.e., the crystal structure residue number 46 was shown at first position. The residue numbering is presented accordingly in all the RMSF results.

## 3. Results and Discussion

### 3.1. Pharmacophore Modeling and Validations

The ligand based pharmacophore design was based on known actives possessing pharmacological activity over the selected target. The pharmacophore model was developed by considering 69 agonists with known IC50 values as a dataset [27,39]. IC50 values of the selected molecules were converted to pIC50 and further dataset was divided into two groups, training and test sets, respectively. Twenty-two molecules with pIC50 > 3.5 were considered as a training set for the generation of pharmacophore models and the remaining molecules were considered as a test set. By keeping the maximum and minimum number of sites option to four in pharmacophore hypothesis application settings, a total of six hypotheses with altered variant combinations were generated. Based on survival score and superior alignment with actives, the best top hypothesis AADHR was selected. The scoring function, which was employed to rank generated variants, was determined based on the number of sites and ligands matched, vector size, selectivity, and volume size, which would further help in the selection of the hypothesis rationally.

**Figure 1:**
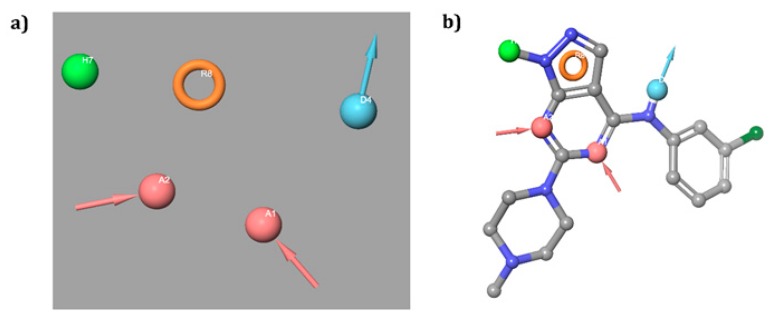
(**a**) Hypothesis AADHR generated from pharmacophore modeling highlighting intra distances; (**b**) Alignment of hypothesis against molecule in actives dataset.

The five sites hypothesis AADHR revealed highest survival score of 5.143 (Table 1) comprising of two hydrogen bond acceptors (A), one donor (D), one hydrophobic group (H) and a ring aromatic (R) feature. The hypothesis and its alignment against molecule in the actives dataset were depicted in the Figure 1. The hypothesis showed an EF of 44, the count and percentage of actives in the top 20% of the decoy results was 82.6%, respectively. The scores represent pharmacophore hypothesis competence, adequacy and capability of screening best number of actives from database.

### 3.2. Screening of the ZINC Natural Molecule Database

Advanced pharmacophore screening of phase module was used for screening the built and minimized 3D ZINC natural database. The database was screened with generated hypothesis by retaining default parameters except for matching tab, which was set to five out of five. Using the fitness score >1.0, the top-listed 1000 hits were taken into account and progressed to ADME analysis using QikProp application. Eight ligand molecules resulted as best hits based on CNS activity with +2, followed by zero violation of the Lipinski’s rule of five and good QPlogBB range in addition to approved PAINS filter (Table 2). Eight ligand molecules were carried forward to docking studies to analyze their binding affinity with the 5HT_2C_ receptor.

### 3.3. Receptor-Ligand Docking Studies (5HT_2C_)

Receptor downloaded from protein databank was prepared using the protein preparation wizard to convert the raw structure into a refined structure [40,41]. Through a receptor grid generation step, the prepared protein active site was locked and docked with seven out of eight ligand compounds selected from the above steps. ZINC40312983 and ZINC40312987 were isomers and, hence, only ZINC40312983 was considered for docking studies using the Glide XP docking protocol. Along with seven hits, the standard agonist drug Lorcaserin was also added to the docking list for comparative analysis and their docking G-scores were found ranging between −9.58 to −6.26 (Table 2). Based on the molecules hydrogen bond interactions with the conserved amino acid Asp 134 of the receptor, five ligand molecules were selected and their interaction profiles along with Lorcaserin were studied.

**Figure 2:**
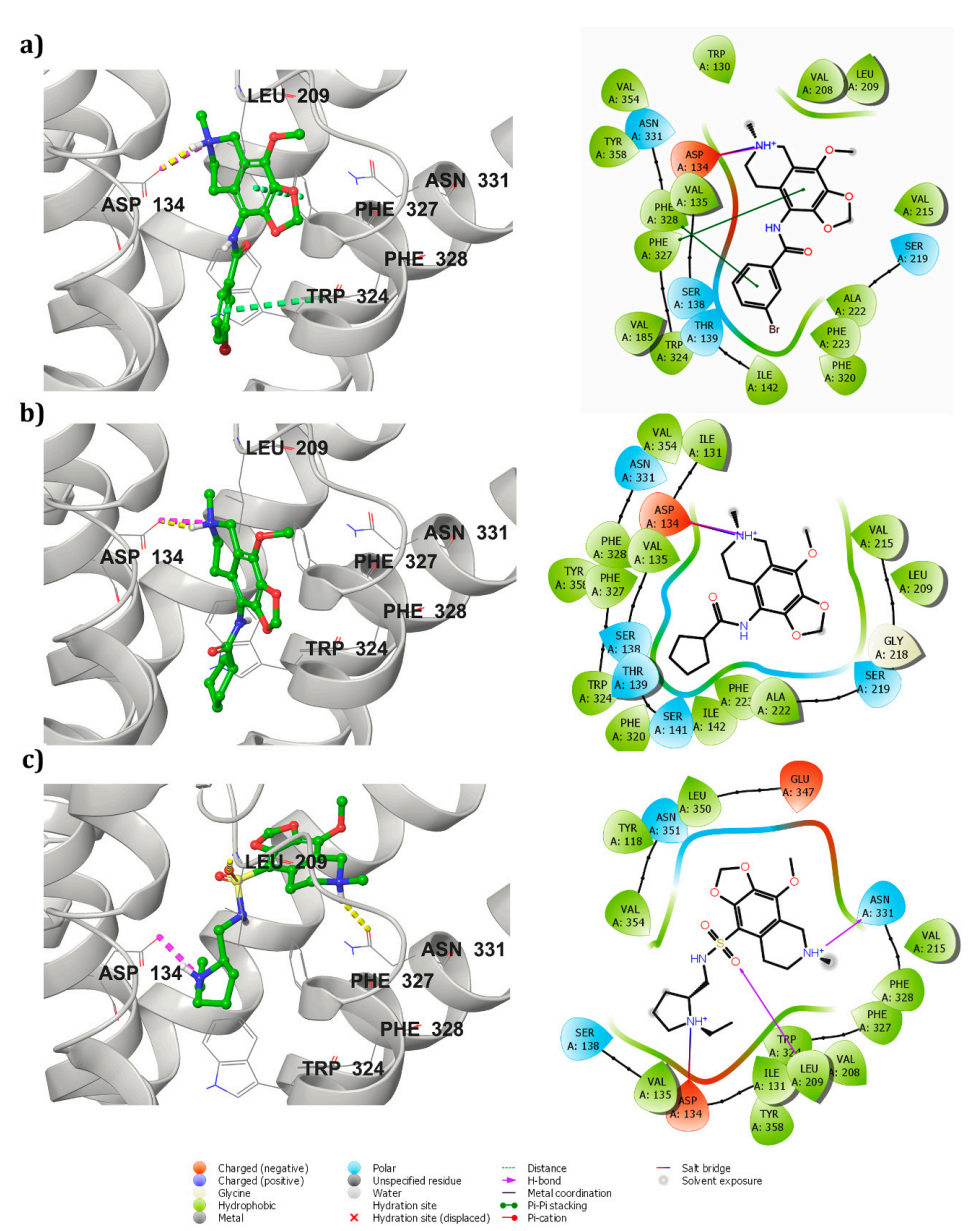
Interaction profile of molecules with 5HT_2C_ receptor after docking studies depicted in both 3D (left side: Hydrogen bonds (yellow), salt bridge (magenta), and π–π (green) interactions as shown in dotted lines) and 2D (right side) forms (**a**) 5HT_2C_ receptor-Zinc32123870 complex, (**b**) 5HT_2C_ receptor-ZINC32124535 complex, and (**c**) 5HT_2C_ receptor-Zinc40312983 complex.

The known drug Lorcaserin maintained three interactions, namely one hydrogen bond (NH2+ with =O of Asp 134 residue), one salt bridge (NH2+ with the oxygen atom of Asp 134 residue), and one π–π interaction with Phe 327 residue, in the active site of the receptor (Supplementary Figure S1). The receptor–ZINC32123870 in complex form was sustained by two π–π stackings, one hydrogen bond and a salt bridge. One hydrogen bond was formed between a NH+ group of ZINC32123870 with the oxygen atom of important amino acid residue Asp 134 of the receptor and salt bridge was also perceived with the same amino acid. Among two π–π interactions, one was maintained between Phe 327 of receptor with an aromatic group of main moiety and another one was found between Phe 328 and aromatic ring present in the R-group of ligand Figure 2a. ZINC32124535 made one hydrogen bond and one salt bridge with receptor by the end of the docking studies. The two interactions were shared between the oxygen atom of Asp 134 and NH+ of ligand (main moiety), as seen in Figure 2b. The binding affinity between receptor-ZINC40312983 complex (Figure 2c) was attributed to one salt bridge and two hydrogen bonds. The interactions of the molecule with receptor are as follows: Oxygen atom of Asp 134 and NH+ of ZINC40312983 molecule (salt bridge), NH+ (main moiety) of the molecule with Asn 331(=O) and =O attached to the sulphur atom in the molecule with NH group of the Leu 209 (hydrogen bonds).

Receptor and ZINC32123898 complex was maintained through a salt bridge and two π–π interactions. The salt bridge was formed between ligand (main moiety NH+ group) and oxygen atom of Asp 134 amino acid of the receptor. Two stackings were observed with the Phe 328 residue: One with a five-membered ring and another with a six-membered ring of main moiety. The docking studies of receptor and ZINC32123782 complex revealed one hydrogen bond (NH+) and one salt bridge (NH+) interaction with Asp 134. Along with them, three π–π interactions occurred, one with Phe 328 second Trp 324 with aromatic ring present in the R-group of ligand and the third π–π interaction was shared between aromatic ring (main moiety) of ligand with Phe 327.

The interaction of the highly conserved residue Asp 134 with molecules was considered the most important in performing agonist activity [27,39,41,42]. The other important amino acids in the vicinity region reported were Ser 138, Tyr 358, Tyr 324, and Arg 195 [43,44]. The standard drug and five screened hits showed interactions with these important amino acids in the active site vicinity. The important residue Asp 134 was found to maintain an interaction with the NH+ group of the compounds either through hydrogen bond or salt bridge. Among these five compounds, three compounds (ZINC32123870, ZINC40312983 and ZINC32124535) individually produced one hydrogen bond and one salt bridge. The rest of the two established only one salt bridge with Asp 134. These interactions resulted because the NH+ group of compounds protruded toward Asp 134 residue during docking and thereby attained a stable confirmation inside active site. In addition, the attractive forces of negatively charged Asp residue and positively charged NH+ group of hits assisted them to accommodate well inside active site pocket by forming a stable complex.

### 3.4. Dynamic Simulations of the 5HT_2C_-Ligand Complexes

**Figure 3:**
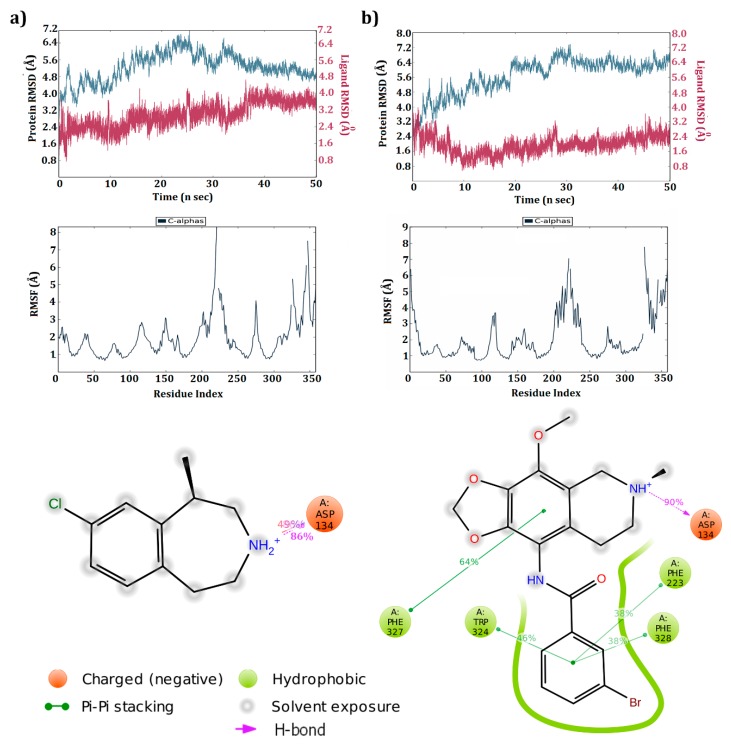
The 5HT_2C_ receptor and molecules complex deviation, fluctuations, and interaction percentage at the end of simulation study: (**a**) Receptor—Lorcaserin; (**b**) Receptor—ZINC32123870.

Based on docking studies, five ligand molecules were found to produce interactions with active site amino acids. To verify steadiness of these interactions, the 5HT_2C_-ligand complexes were further subjected to molecular dynamic simulations for a period of 50 ns. Each molecule’s deviation, fluctuation, and percentage of H-bond interactions were analyzed. The known drug Lorcaserin was found to occupy the active pocket by maintaining a strong hydrogen bond with Asp 134 (97%). The receptor in the presence of Lorcaserin was found to produce deviations between 2.75–6.3 Å until 25 ns, where the deviation declined to 4.8 Å which continued until the end of the simulation (Figure 3a). The majority of ligand deviations were observed between 2.4 to 3.2 Å and from 40ns, a steady pace in deviations was observed till the end of simulations. The majority of amino acids fluctuated below 3.0 Å and a few amino acids (31 and 160) fluctuated above 4.5 Å.

At the end of simulations run, only three complexes were found to maintain the hydrogen bond with the conserved amino acid of the receptor. ZINC32123870 molecule retained all interactions produced in the docking studies. Along with them, two more π–π interactions were observed with Trp 324 and Phe 223 at the end of simulations. The salt bridge formed during the docking studies with Asp134 vanished by the end of simulations (Figure 3b). The receptor made huge deviations, ranging from 2.4 to 7.1 Å in complex with ZINC32123870. The inclinations in deviations were reported up to 30 ns, reaching a maximum deviation of 7.1 Å. From there, deviations declined, ranging between 6.0 to 7.0 Å. In contrast, ligand deviations declined from 3.2 to 0.8 Å at the end of 10 ns and deviations later increased, with the majority of them observed between 2.0 to 3.0 Å. With regard to amino acid fluctuations during simulation, most of the amino acids were reported below 3.0 Å except for two regions (one between 200–240 and the other in the tail-end region).

**Figure 4:**
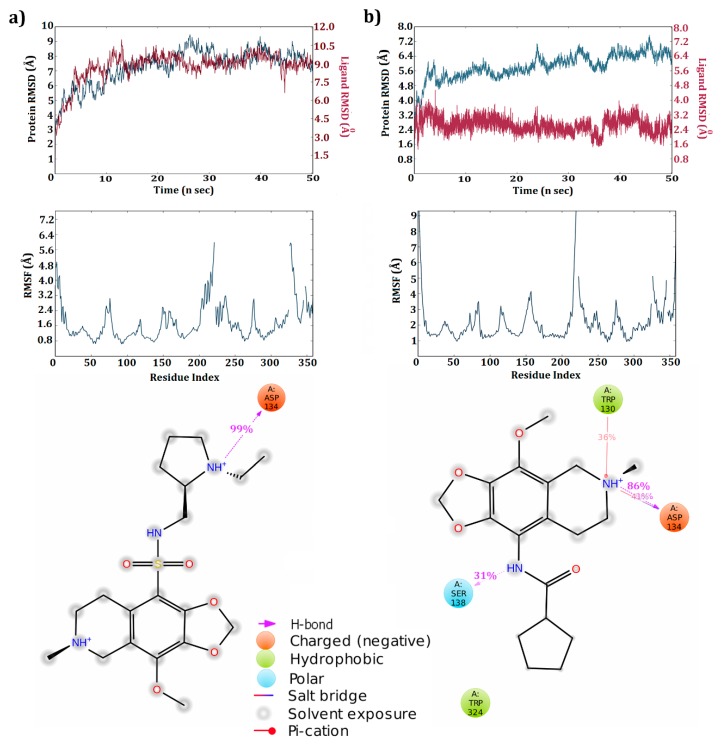
Deviations, fluctuations, and interaction percentages between the 5HT_2C_ receptor and molecules at the end of 50 ns simulation run: (**a**) 5HT_2C_—ZINC40312983; (**b**) 5HT_2C_—ZINC32124535.

The receptor-ZINC40312983 complex generated only one H-bond with Asp 134 (99%) at the end of simulations (Figure 4a). Major changes observed after simulation was conversion of salt bridge between molecule and Asp 134 to hydrogen bond and all remaining interactions observed in docking study were vanished after simulation. The RMSD graph of complex obtained at the end of simulations reported that protein made huge deviations, inclinations, and declinations throughout the simulation within a range of 3.5–9.0 Å. Concomitantly, ligand deviations during the simulation run were observed to be high, ranging between 3.0 and 10.5 Å. In the complex state, both ligand and the receptor maintained nearly the same pace of deviations graph throughout the simulations. The RMSF graph reported that most of amino acids fluctuations were below 3.0 Å. Amino acids between 200–225 and around 325 fluctuated more during the simulation run and were reported above 3.0 Å.

The ZINC32124535 molecule maintained similar interactions produced during docking studies with Asp 134 even after simulation run completion (Figure 4b). Protein deviations were reported from 3.2 Å to a maximum of 7.2 Å and finally settled below 7.2 Å. Compared to protein, ligand maintained steady state deviations between 2.4 to 3.2 Å throughout the simulations. The tail-end amino acids fluctuated more during simulations and were reported above 3.0 Å, while the majority of amino acids fluctuated below 3.0 Å. The receptor residues in the 200–225 range reported deviations above an acceptable range (1–3 Å).

In all the 5HT_2C_ receptor-ligand complexes, residues ranging 200–240 produced fluctuations and were majorly loop regions. Generally, loop regions fluctuated more during simulations to attain a stable conformational state. Deviations made by the receptor during the simulation runtime inform us about the structural conformational changes undergone by residues in the protein for attaining a stable confirmation. In all the four complexes, the 5HT_2C_ receptor produced deviations throughout the simulation run. The receptor and molecules also generated deviations. This confirms that molecule conformational changes are proportional to protein conformational changes. Moreover, molecules maintained their position inside the active pocket supported with strong interactions with residues in the active pocket.

The major difference in binding modes observed at the end of simulations was mainly because of the flexible nature adopted by the receptor and molecules during simulations. In general docking studies, only the ligand is in flexible mode, while the receptor is in rigid mode. This major difference in the flexibility natures maintained by the receptor and ligand during docking and simulation studies plays a crucial role in generating interactions between the receptor and ligand. The flexibile nature supports both the receptor and ligand in generating a higher number of conformational states and thereby aids in the development of new hydrogen bonds or deduction of existing hydrogen bonds.

In the present study, at the end of simulation only three molecules, ZIN32123870, ZINC40312983 and ZINC32124535 were found to maintain a strong hydrogen bond interaction with the active site amino acid Asp 134. After simulations, the other four molecules failed to conserve the interactions made during the docking studies. The simulation analysis of three complexes confirm that the interactions made by the molecules with the crucial residue of the receptor was conserved throughout simulation time and the percentage of H-bond interaction between molecules and Asp134 was above 85%. These results reinstate that the molecule was stable inside the active pocket with the same binding profile. Receptor deviations in all the complexes were mainly due to flanking end regions. Counteracting these deviations, ligands also showed certain deviations without losing the H-bond interactions or falling out of the active pocket.

### 3.5. Cross Validation of the Hits

Molecular specificity against the target is more important to reduce adverse side effects. Specificity plays a crucial role in screening agonists targeting the 5HT_2C_ receptor because of the high sequence similarity of the conserved domains, as well as the overall sequence identity among the family members (55% with 5HT_2A_ and 58% with 5HT_2B_ receptors, respectively) [41]. The three best hits identified against 5HT_2C_ receptor, ZINC32123870, ZINC40312983, and ZINC32124535, were cross validated against other members of the family, 5HT_2A_ and 5HT_2B_ receptors, using the same protocol along with Lorcaserin.

#### 3.5.1. 5HT_2A_ Receptor

The 5HT_2C_ receptor crystal structure (PDB ID: 6BQG) was chosen as a template for developing the 3D structure of 5HT_2A_ receptor. The energy-based model was built and validated using Ramachandran plot and Errat score. The Ramachandran plot summarized that the majority of amino acids were in the allowed region (99.7%) with a few outliners (0.3%). However, the ERRAT score of the built model showed an overall quality factor of 83.11, which revealed that the model needed refinement. The model refinement was carried out using the Desmond module with default parameters. After refinement, the model was grouped into five clusters based on deviations and energy using a clustering method. The best cluster center, based on the minimal deviations and energy was considered for further docking and simulation studies.

**Figure 5:**
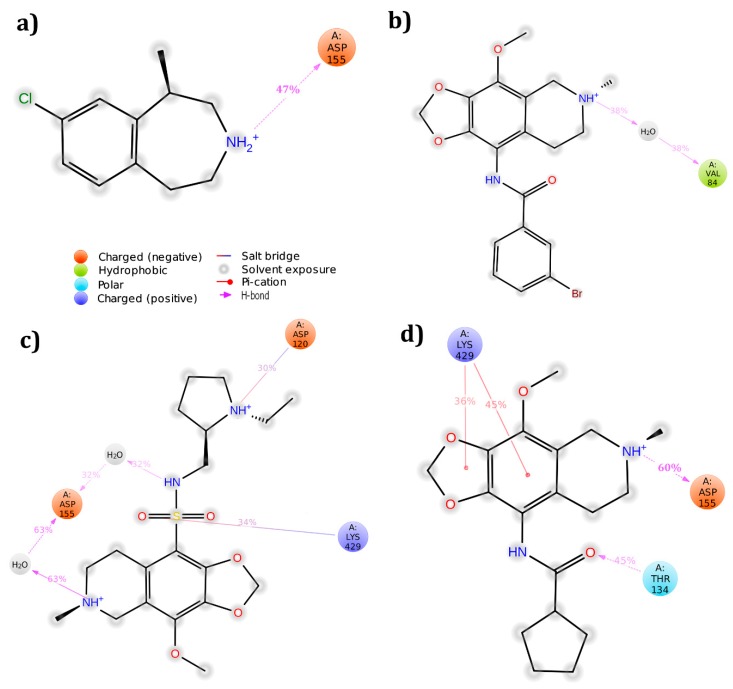
Binding modes of molecules with 5HT_2A_ receptor after simulation studies: (**a**) Lorcaserin; (**b**) ZINC32123870; (**c**) ZINC40312983; and (**d**) ZINC32124535.

Using the Sitemap module, the active pocket in the receptor was identified and the grid was generated around the site through receptor grid generation application. The three hit molecules were docked into this active site using Glide XP docking protocol and their interactions were analyzed. None of the molecules produced interactions with the conserved residue at the end of docking study. However, to further analyze the interaction profile, the three complexes were simulated. ZINC32124535 molecule was found to produce hydrogen bond interaction with conserved residue Asp 155 (60%), whereas the other two molecules failed to produce hydrogen bonds with any one of the residues in active pocket (Figure 5). After simulation, the PRIME energies were also calculated. ZINC32123870 molecule showed the highest energy, followed by ZINC32124535 and ZINC40312983 (Table 3).

#### 3.5.2. 5HT_2B_ Receptor

**Figure 6:**
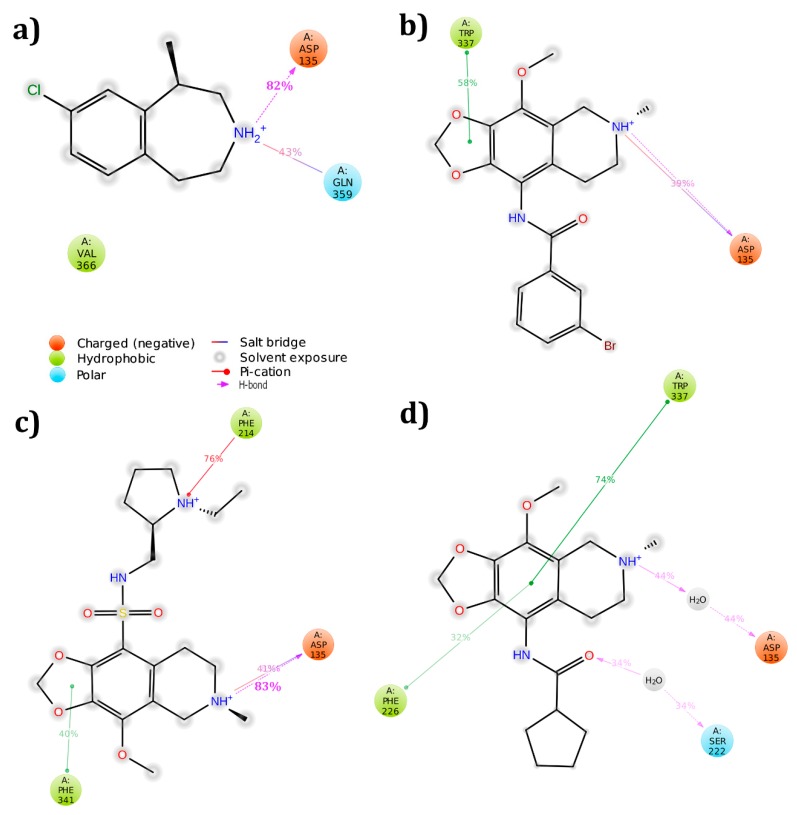
5HT_2B_ receptor amino acids interaction profiles with molecules after completion of simulation run: (**a**) Lorcaserin; (**b**) ZINC32123870; (**c**) ZINC40312983; and (**d**) ZINC32124535.

The receptor crystal structure was imported from protein databank (PDB ID: 4IB4) with a resolution of 2.7 Å. All three hit molecules produced interactions with active pocket amino acids along with the highly conserved amino acid Asp 135. Further, complexes were subjected to molecular dynamic simulations to verify whether interactions were retained or lost after 50 ns run time. Except for the 5HT_2B_-ZINC32124535 complex, the remaining all complexes preserved interactions with the conserved amino acid (Figure 6). The PRIME energy values after simulation were also listed. The ZINC32124535 molecule showed the highest energy, followed by ZINC32123870 and ZINC40312983 (Table 3).

### 3.6. Homology Model vs. Crystal Structure

Until the crystal structure was made available, the majority of the screening of novel molecules against the 5HT_2C_ receptor were carried with homology models built using the bovine rhodopsin and GPCR’s (G protein-coupled receptors) like human β2-adrenergic G protein-coupled receptor. The accuracy of the built model is considered very important for generating a potential hit against the target. If it has flaws, it leads to the generation of false positives. Hence, there is also a need to revalidate the reported potential hits with the crystal structure. To elucidate the difference in the docking and simulation studies with regard to the homology model and crystal structures, this study implemented the homology model vs. crystal structure with the selected hits. 

Homology modeling of 5HT_2C_ was performed using a prime homology modeling tool. The X-ray crystal structure of 5HT_2B_ receptor (PDB ID: 4NC3 with a resolution 2.8 Å) with 53.825% similarity against query sequence was preferred as a template for developing a 3D structure of the receptor. The Ramachandran plot for the built model reported 99.7% amino acids of the predicted model were in allowed regions and 0.3% were in disallowed regions. Molecular dynamic simulations were carried with the modeled 5HT_2C_ receptor to further refine the built model for a time period of 50 ns. The refined model Ramachandran plot reported 85.4% amino acids in the favorable region, 13.9% in the additionally allowed region, and 0.6% in generously allowed regions, with no amino acids in disallowed regions.

**Figure 7:**
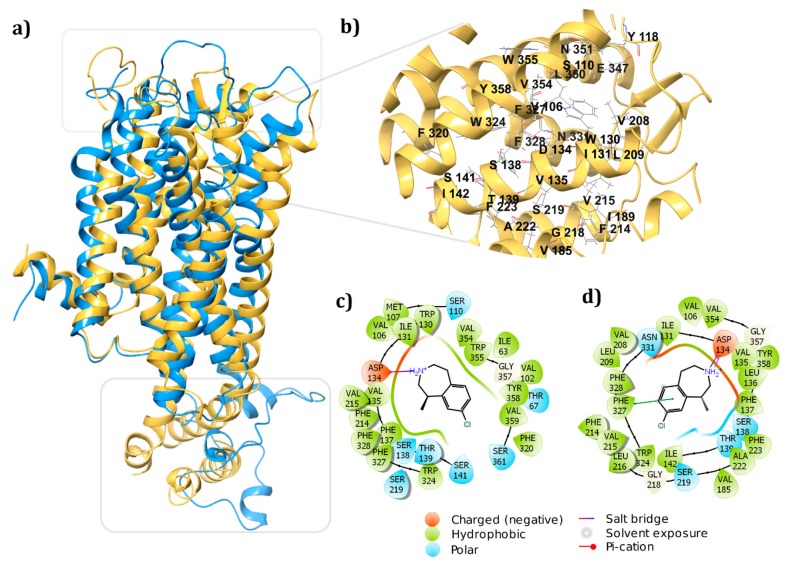
(**a**) Superimposition of homology model (blue colored) with crystal structure (PDB ID: 6BQG; brown colored), major deviations were highlighted inside box. (**b**) Influential and important amino in active pocket of 5HT_2C_ receptor; (**c**) LigPlot of 5HT_2C_ (homology model) with the known drug Lorcaserin; (**d**) LigPlot of 5HT_2C_ (crystal structure) with the known drug Lorcaserin.

The crystal and modeled protein structures were superimposed to check major deviations between their structures with regard to the active pocket. Two regions, namely 185–215 and 255–300 amino acid residue stretches in built model, were loops, whereas the crystal structure had helices along with a loop with regard to the 185–215 stretch, and the same 255–300 stretch replaced with BRIL [40] was majorly in helix form (Figure 7a). The major modifications (185–215) made an influential impact on the distances and bond angles of the amino acids (Figure 7b) in the active pocket, which was clearly observed in docking studies of receptor (modeled and crystal structure) with the standard drug lorcaserin (Figure 7c,d). The docking G score with the homology model was −4.66 while the crystal structure exhibited a G score of −6.55. Molecular dynamic simulation studies showed one hydrogen bond and salt bridge interaction with Asp 134 of both the homology modeled and crystal structure 5HT_2C_ receptor. The H-bond was shared between NH+ group (main moiety) of Lorcaserin with C=O of Asp 134 residue of the receptor. In addition, only the crystal 5HT_2C_ receptor- Lorcaserin complex showed π–π interaction. The π–π interaction was observed between Phe 327 residue and with 3-methylazepane ring (six-membered ring) of Lorcaserin.

From the cross validation results, out of three hits, ZINC32124535 was found to be best potential hit against the 5HT_2C_ receptor. Even though it showed interaction with the 5HT_2A_ receptor conserved region, considering the percentage of hydrogen bond interactions against the conserved residue, the molecule was more specific toward the 5HT_2C_ receptor. The other two hits also made interactions with conserved amino acid of two receptors (5HT_2C_ and 5HT_2B_, respectively), with a difference in the hydrogen bond percentage. Comparing the known drug interaction among the three receptors, the hydrogen bond interaction percentage after simulations studies specifies that the hit is more specific to 5HT_2C_ receptor. From these observations, all three molecules were concluded as the potential hits against the 5HT_2C_ receptor. Based on interaction percentages and cross validation results, out of three hits, ZINC32124535 was found to be best potential hit against the 5HT_2C_ receptor, followed by ZINC40312983 and ZINC32123870.

## 4. Conclusions

Obesity has become a life-threatening multifactorial global pandemic. Its economic burden compels us to look for more effective measures, as current measures such as lifestyle modifications, bariatric surgery or medications/supplements available in market are not sufficient to control the disease. Therefore, there is a need to develop new drugs for the treatment of obesity. Among the targets reported so far, the 5HT_2C_ receptor has gained increased attention and is also a challenging target because of high sequence similarity of other receptors within the group. This study mainly focused on elucidating the specificity of screened molecules among 5HT_2A_, 5HT_2B_, and 5HT_2C_ receptors. Overall, the present computational study reported three molecules, ZINC40312983, ZINC32123870, and ZINC32124535, to be the best agonists for the 5HT_2C_ receptor compared to other members in this subtype. ZINC32124535 was identified as the best potential hit and needs further in vitro and in vivo validations.

## Figures and Tables

**Table 1:** Hypothesis generated through PHASE run.

Hypothesis ID	No. of Sites	Survival Score	Site Score	Vector Score	Volume Score	Selectivity
AADHR	5	5.143	0.766	0.825	0.622	1.515
AAADH		4.909	0.688	0.769	0.517	1.214
AAAH	4	5.064	0.957	0.994	0.650	1.015
AAHR		5.171	0.860	0.972	0.631	1.217
DHRR		5.090	0.660	0.934	0.592	1.490
ADHR		4.939	0.737	0.919	0.581	1.287

**Table 2:** Docking scores of potential hits and their pharmacological properties.

Core Moiety	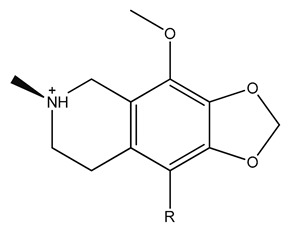							
Molecule	R	G-Score	PAINS	CNS ^a^	mol_MW ^b^	QPlog BB ^c^	Percent Human Oral Absorption ^d^	Rule of Five ^e^
ZINC32123898	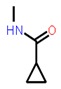	−9.58	Passed	2	304.34	0.475	92.5	0
ZINC32123782	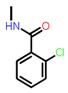	−8.93	Passed	2	374.82	0.499	96.8	0
ZINC32123870	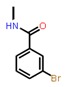	−8.66	Passed	2	419.27	0.457	95.7	0
ZINC32124535	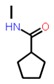	−8.51	Passed	2	332.39	0.476	96.2	0
ZINC40312983	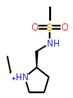	−7.79	Passed	2	411.51	0.539	74.3	0
ZINC40309640	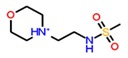	−7.80	Passed	2	413.48	0.462	66.3	0
ZINC32124107	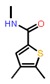	−6.26	Passed	2	374.45	0.472	100	0
Lorcaserin	-	−6.55	Passed	2	181.58	0.483	100	0

^a^ Predicted central nervous system activity on –2 (inactive) to +2 (active) scale. ^b^ Molecular weight of the molecules ranging 130.0–725.0. ^c^ Predicted brain/blood partition coefficient ranging from –3.0 to 1.2, ^d^ Predicted human oral absorption percentage considering >80% as high <25% as poor, ^e^ Lipinski’s rule of five violations maximum considered is four.

**Table 3:** Interactions of three potential hits along with known drug during the docking and simulation studies along with PRIME energy calculations.

Molecule	Receptor	Before MDS		After MDS	
		Interactions Profile	PRIME Energy (Kcal/mol)	Interactions	PRIME Energy (Kcal/mol)
**Lorcaserin**	5HT_2C_	Asp134(1HB & 1SB) Phe327(π–π)	−41.27	Asp134(1HB)	−43.72
	5HT_2B_	Asp135(1SB)	−43.24	Asp135(1HB) Gln359(1SB)	−42.04
	5HT_2A_	Ser 432	−32.48		−33.01
ZINC 32123870	5HT_2C_	Asp134(1HB & 1SB) Phe327(π–π), Phe328(π–π)	−58.30	Asp134(1HB) Phe327(1HB) Phe223(π–π) Trp324(π–π) Phe328(π–π)	−76.03
	5HT_2B_	Asp135(1HB & 1SB) Phe340(pi-cation)	−62.85	Asp135(1HB & 1SB),Trp337(π–π)	−64.20
	5HT_2A_	Ser 432(1HB)	−44.62	No interactions	−58.27
ZINC 40312983	5HT_2C_	Asp134(1SB) Leu209(1HB) Asn331(1HB)	−52.52	Asp134(1HB) Tyr358(pi-cation) Ser110(1HB)	−34.74
	5HT_2B_	Asp135(1HB & 1SB) Phe340(pi-cation)	−62.56	Asp135(1HB & 1SB) Phe214(pi-cation) Phe341(π–π)	−63.55
	5HT_2A_	Lys 429(1HB)	−48.20	Asp120(pi-cation)	−31.12
ZINC 32124535	5HT_2C_	Asp134(1HB & 1SB)	−49.66	Asp134 (1HB & 1SB)	−70.54
	5HT_2B_	Asp135(1SB)	−55.75	Phe226(π–π) Trp337(π–π)	−70.24
	5HT_2A_	Lys 429 (1HB&2pi-cation)	−38.39	Asp155(1HB) Thr134(1HB) Lys 429(2pi-cation)	−41.34

## Supplementary Materials

The following are available online at https://www.mdpi.com/2218-273X/9/10/556/s1, Figure S1: Interaction profile of known drug lorcaserin with 5HT_2C_ receptor after docking studies depicted in both 3D (left side; Hydrogen bonds (yellow), salt bridge (magenta) and π–π (green) interactions as shown in dotted lines) and 2D (right side) forms.

## References

[1] Kopelman P.G. (2000). Obesity as a medical problem. Nature.

[2] Jebb S. (2004). Obesity: Causes and consequences. Women’s Health Med..

[3] Finer N. (2006). Medical consequences of obesity. Medicine.

[4] Schwartz M.W., Seeley R.J., Zeltser L.M., Drewnowski A., Ravussin E., Redman L.M., Leibel R.L. (2017). Obesity pathogenesis: An Endocrine Society scientific statement. Endocr. Rev..

[5] Neeland I.J., Poirier P., Després J.-P. (2018). Cardiovascular and metabolic heterogeneity of obesity: Clinical challenges and implications for management. Circulation.

[6] Gadde K.M., Apolzan J.W., Berthoud H.-R. (2018). Pharmacotherapy for patients with obesity. Clin. Chem..

[7] Cohen J.B., Gadde K.M. (2019). Weight Loss Medications in the Treatment of Obesity and Hypertension. Curr. Hypertens. Rep..

[8] Tecott L.H., Sun L.M., Akana S.F., Strack A.M., Lowenstein D.H., Dallman M.F., Julius D. (1995). Eating disorder and epilepsy in mice lacking 5-HT2c serotonin receptors. Nature.

[9] Millan M.J., Peglion J.-L., Lavielle G., Perrin-Monneyron S. (1997). 5-HT2C receptors mediate penile erections in rats: Actions of novel and selective agonists and antagonists. Eur. J. Pharmacol..

[10] Delgado P.L., Moreno F.A. (1998). Hallucinogens, serotonin and obsessive-compulsive disorder. J. Psychoact. Drugs.

[11] Martin J., Bös M., Jenck F., Moreau J.-L., Mutel V., Sleight A., Wichmann J., Andrews J., Berendsen H., Broekkamp C. (1998). 5-HT2C receptor agonists: Pharmacological characteristics and therapeutic potential. J. Pharmacol. Exp. Ther..

[12] Weissman N. (1999). Appetite suppressant valvulopathy: A review of current data. Cardiovasc. Rev. Rep..

[13] Roth B.L., Lopez E., Patel S., Kroeze W.K. (2000). The multiplicity of serotonin receptors: Uselessly diverse molecules or an embarrassment of riches?. Neuroscience.

[14] Shapiro D.A., Roth B.L. (2001). Insights into the structure and function of 5-HT2 familyserotonin receptors reveal novel strategies for therapeutic target development. Expert Opin. Ther. Targets.

[15] Rocha B.A., Goulding E.H., O’Dell L.E., Mead A.N., Coufal N.G., Parsons L.H., Tecott L.H. (2002). Enhanced locomotor, reinforcing, and neurochemical effects of cocaine in serotonin 5-hydroxytryptamine 2C receptor mutant mice. J. Neurosci..

[16] Chou-Green J.M., Holscher T.D., Dallman M.F., Akana S.F. (2003). Compulsive behavior in the 5-HT2C receptor knockout mouse. Physiol. Behav..

[17] Isaac M. (2005). Serotonergic 5-HT2C receptors as a potential therapeutic target for the design antiepileptic drugs. Curr. Top. Med. Chem..

[18] Miller K.J. (2005). Serotonin 5-ht2c receptor agonists: Potential for the treatment of obesity. Mol. Interv..

[19] Rashid M., Manivet P., Nishio H., Pratuangdejkul J., Rajab M., Ishiguro M., Launay J.-M., Nagatomo T. (2003). Identification of the binding sites and selectivity of sarpogrelate, a novel 5-HT2 antagonist, to human 5-HT2A, 5-HT2B and 5-HT2C receptor subtypes by molecular modeling. Life Sci..

[20] Girardet C., Butler A.A. (2014). Neural melanocortin receptors in obesity and related metabolic disorders. Biochim. Biophys. Acta (BBA) Mol. Basis Dis..

[21] Martin C.K., Redman L.M., Zhang J., Sanchez M., Anderson C.M., Smith S.R., Ravussin E. (2011). Lorcaserin, a 5-HT2C receptor agonist, reduces body weight by decreasing energy intake without influencing energy expenditure. J. Clin. Endocrinol. Metab..

[22] Blundell J.E., Halford J.C. (1998). Serotonin and appetite regulation. CNS Drugs.

[23] Fitzgerald L.W., Ennis M.D. (2002). 5-HT2C receptor modulators: Progress in development of new CNS medicines. Annu. Rep. Med. Chem..

[24] Smith S.R., Prosser W.A., Donahue D.J., Morgan M.E., Anderson C.M., Shanahan W.R., Group A.S. (2009). Lorcaserin (APD356), a selective 5-HT2C agonist, reduces body weight in obese men and women. Obesity.

[25] Bohula E.A., Wiviott S.D., McGuire D.K., Inzucchi S.E., Kuder J., Im K., Fanola C.L., Qamar A., Brown C., Budaj A. (2018). Cardiovascular safety of lorcaserin in overweight or obese patients. N. Engl. J. Med..

[26] DiNicolantonio J.J., Chatterjee S., O’Keefe J.H., Meier P. (2014). Lorcaserin for the treatment of obesity? A closer look at its side effects. Arch. Dis. Child..

[27] Ahmed A., Choo H., Cho Y.S., Park W.-K., Pae A.N. (2009). Identification of novel serotonin 2C receptor ligands by sequential virtual screening. Bioorg. Med. Chem..

[28] Dixon S.L., Smondyrev A.M., Knoll E.H., Rao S.N., Shaw D.E., Friesner R.A. (2006). PHASE: A new engine for pharmacophore perception, 3D QSAR model development, and 3D database screening: 1. Methodology and preliminary results. J. Comput. Aided Mol. Des..

[29] Veeramachaneni G.K., Raj K.K., Chalasani L.M., Annamraju S.K., JS B., Talluri V.R. (2015). Shape based virtual screening and molecular docking towards designing novel pancreatic lipase inhibitors. Bioinformation.

[30] Baell J.B., Holloway G.A. (2010). New substructure filters for removal of pan assay interference compounds (PAINS) from screening libraries and for their exclusion in bioassays. J. Med. Chem..

[31] Altschul S.F., Gish W., Miller W., Myers E.W., Lipman D.J. (1990). Basic local alignment search tool. J. Mol. Biol..

[32] Berman H., Westbrook J., Feng Z., Gilliland G., Bhat T., Weissig H., Shindyalov I., Bourne P. (2000). The protein data Bank nucleic acids research. Nucleic Acids Res..

[33] Sastry G.M., Adzhigirey M., Day T., Annabhimoju R., Sherman W. (2013). Protein and ligand preparation: Parameters, protocols, and influence on virtual screening enrichments. J. Comput. Aided Mol. Des..

[34] Jorgensen W.L., Maxwell D.S., Tirado-Rives J. (1996). Development and testing of the OPLS all-atom force field on conformational energetics and properties of organic liquids. J. Am. Chem. Soc..

[35] Friesner R.A., Murphy R.B., Repasky M.P., Frye L.L., Greenwood J.R., Halgren T.A., Sanschagrin P.C., Mainz D.T. (2006). Extra precision glide: Docking and scoring incorporating a model of hydrophobic enclosure for protein—Ligand complexes. J. Med. Chem..

[36] Genheden S., Ryde U. (2015). The MM/PBSA and MM/GBSA methods to estimate ligand-binding affinities. Expert Opin. Drug Discov..

[37] Guo Z., Mohanty U., Noehre J., Sawyer T.K., Sherman W., Krilov G. (2010). Probing the α-helical structural stability of stapled p53 peptides: Molecular dynamics simulations and analysis. Chem. Biol. Drug Des..

[38] Veeramachaneni G.K., Raj K.K., Chalasani L.M., Bondili J.S., Talluri V.R. (2015). High-throughput virtual screening with e-pharmacophore and molecular simulations study in the designing of pancreatic lipase inhibitors. Drug Des. Dev. Ther..

[39] Liu K.K.-C., Cornelius P., Patterson T.A., Zeng Y., Santucci S., Tomlinson E., Gibbons C., Maurer T.S., Marala R., Brown J. (2010). Design and synthesis of orally-active and selective azaindane 5HT2c agonist for the treatment of obesity. Bioorg. Med. Chem. Lett..

[40] Peng Y., McCorvy J.D., Harpsøe K., Lansu K., Yuan S., Popov P., Qu L., Pu M., Che T., Nikolajsen L.F. (2018). 5-HT2C receptor structures reveal the structural basis of GPCR polypharmacology. Cell.

[41] Peng Y., Zhao S., Wu Y., Cao H., Xu Y., Liu X., Shui W., Cheng J., Zhao S., Shen L. (2018). Identification of natural products as novel ligands for the human 5-HT 2C receptor. Biophys. Rep..

[42] Carpenter J., Wang Y., Wu G., Feng J., Ye X.-Y., Morales C.L., Broekema M., Rossi K.A., Miller K.J., Murphy B.J. (2017). Utilization of an active site mutant receptor for the identification of potent and selective atypical 5-HT2C receptor agonists. J. Med. Chem..

[43] Zuo Z., Chen G., Luo X., Puah C., Zhu W., Chen K., Jiang H. (2007). Pharmacophore-directed Homology Modeling and Molecular Dynamics Simulation of G Protein-coupled Receptor: Study of Possible Binding Modes of 5-HT2C Receptor Agonists. Acta Biochim. Biophys. Sin..

[44] Ahmed A., Nagarajan S., Doddareddy M.R., Cho Y.-S., Pae A.-N. (2011). Binding mode prediction of 5-hydroxytryptamine 2C receptor ligands by homology modeling and molecular docking analysis. Bull. Korean Chem. Soc..

