# Supplementary material for: Pharmacophore Directed Screening of Agonistic Natural Molecules Showing Affinity to 5HT2C Receptor
Source: Biomolecules. 2019 Oct 1;9(10):556. doi: 10.3390/biom9100556 (PMC6843766; doi:10.3390/biom9100556)
Supplement: Supplementary file 1 [file biomolecules-09-00556-s001.pdf]

Supplemntary Figures

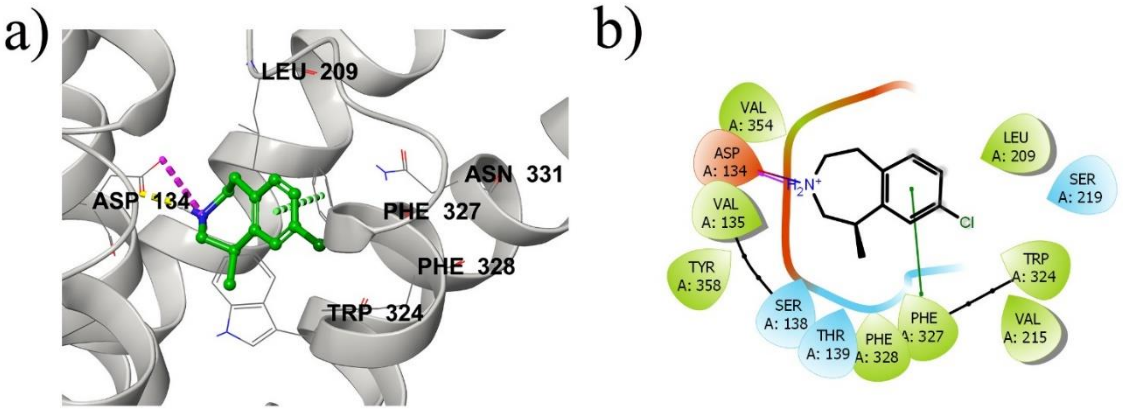

Figure S1: Interaction profile of known drug lorcaserin with 5HT2C receptor after docking studies depicted in both 3D (left side; Hydrogen bonds (yellow), salt bridge (magenta) and π– π (green) interactions as shown in dotted lines) and 2D (right side) forms
